# Adopted neoplastic cells and the consequences of their existence

**DOI:** 10.18632/oncotarget.28408

**Published:** 2023-04-14

**Authors:** Yuri Lazebnik

**Affiliations:** ^1^Lerna Consulting, New Haven, CT 06511, USA

**Keywords:** tumor microenvironment, horizontal oncogenesis, intercellular bridges, cell fusion, cell repair

## Abstract

A view that guides the bulk of cancer research and oncology posits that each neoplastic cell in a tumor is a genetic offspring of another neoplastic cell. Yet, analyzing tumors from transplant patients has revealed that some normal migratory cells adopt the phenotype of neoplastic cells without acquiring their genome, thus becoming what I suggest to call adopted neoplastic cells. This commentary reviews the evidence for the existence of adopted neoplastic cells, outlines the consequences of their presence, and discusses what kind of cells can be adopted, how, and why.

Cancers, or malignant tumors, are defined and diagnosed as tumors whose cells migrate into normal tissues, while benign tumors as those whose cells fail to do so [1]. Why the cells of some tumors migrate and the cells of others do not is not entirely clear, as can be judged by the near absence of drugs that target cancer cell migration [2, 3], by expert opinions that metastasis, the process of migrating to distant organs, is an “almost intractable facet of cancer medicine” [4] in which “attempts to specifically target metastatic pathways have met with near-universal failures” [5], and by the fact that surviving a disseminated cancer is still less likely, and by far, than surviving a round of Russian roulette [5–7], a comparison that is relevant because a cancer patient’s survival is also predicted statistically, that is to say, as a matter of luck.

This situation is not due to the want of effort, as attested by at least 3.3 million articles published on cancer (searching PubMed on March 26, 2023 with ‘cancer [MeSH Major Topic]’ returned 3,374,062 results) since a prominent researcher concluded a century ago that “despite an immense accumulation of data, the solution of the tumor problem waits upon fresh findings” [8]. That this conclusion is still valid reminds us that since it was made some intractable medical problems have been solved not by continuing to accumulate data, but by revisiting or rediscovering neglected observations and models [9–14].

One of the models that are still neglected proposes that tumor cells become migratory not by accumulating genomic aberrations, as a century-old view which dominates cancer research and drug development posits [15–21], but by acquiring this ability from normal migratory cells, like a company that merges with another business to acquire a technology needed to expand into new markets (Figure 1).

**Figure 1:**
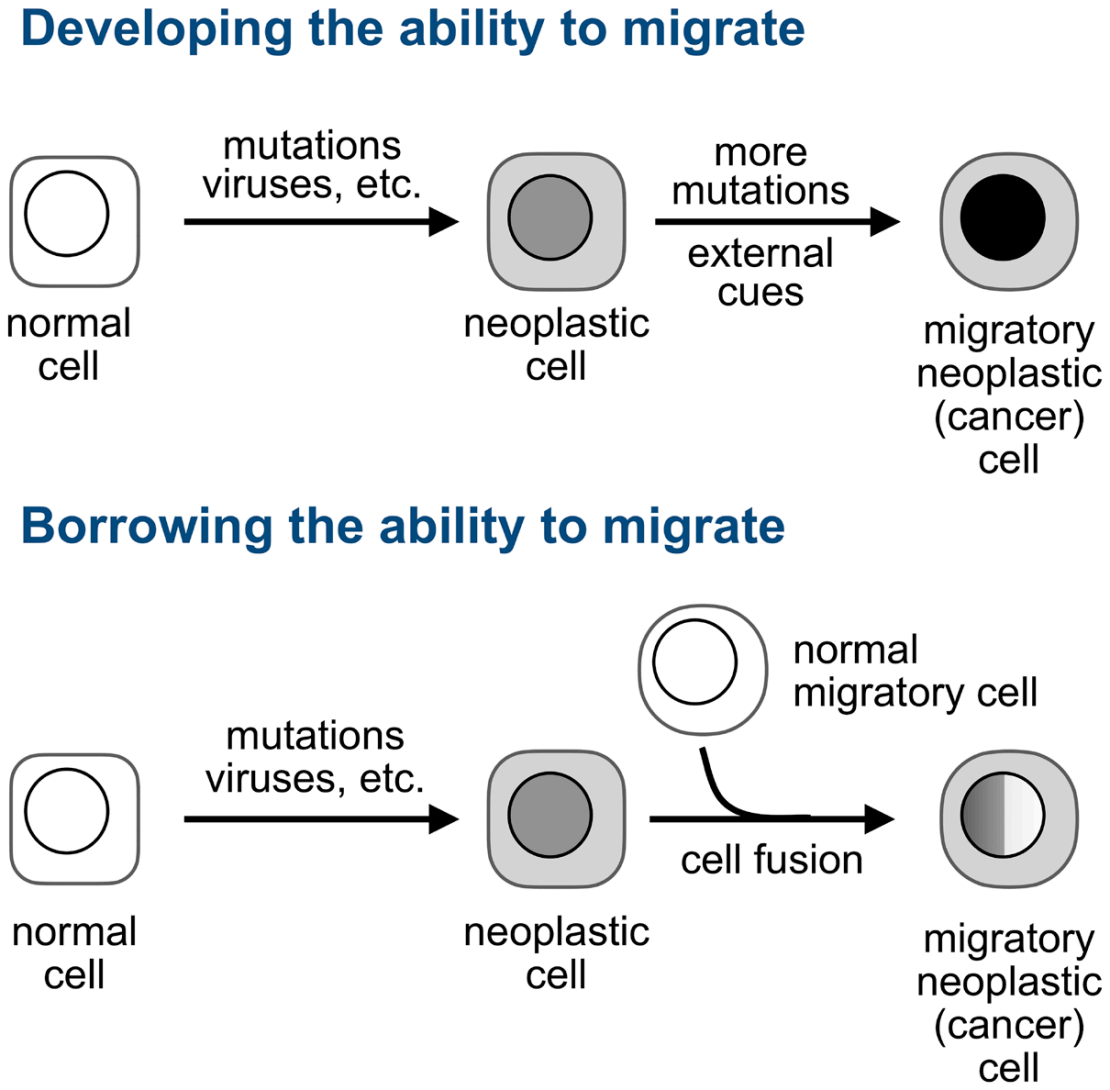
How do tumor cells become migratory? A prevailing view (top) is that tumor cells develop the ability to migrate from within: by acquiring mutations, in response to external cues, or by a combination of these stimuli. An alternative explanations (bottom) is that the ability to migrate is acquired from normal migratory cells. The original hypothesis posited that this acquisition results from fusion between a normal or neoplastic sedentary cell and a normal migratory cell, such as a leukocyte. We will discuss later other modalities of transferring properties from one cell to another.

The original hypothesis, also a century old [22, 23], proposed that the ability of cancer cells to migrate is acquired through cell fusion, a process that combines two or more cells into one by merging their plasma membranes and then combining entire cell contents [24]. The idea was that if a normal cell, or a cell of a benign tumor, fuses to a leucocyte, a migratory cell of the immune system, the resulting hybrids, like cancer cells, would proliferate as one parent and migrate as the other. Also like cancer cells, these hybrids would be diverse because some would “have more the wanderlust of the leukocyte, others more the ability of the somatic cell to function in a sedentary manner,” with additional heterogeneity resulting from the unequal distribution of chromosomes following cell fusion [23].

This hypothesis had laid fallow for six decades, until a serendipitous discovery revealed that human cancers grafted into laboratory animals can become metastatic and evade the immune response by forming hybrids with the cells of the host [25, 26]. Since then, the ability of cell fusion to enable metastases in a variety of animal systems has been confirmed by many studies (reviewed in: [27–30]) and complemented by findings that cell fusion can also produce dormant tumor cells, change their drug sensitivity, suppress the ability to form tumors, which led to the concept of tumor suppressors [31], induce genomic and phenotypic instability [32], affect tumor cell evolution [33, 34], change cell metabolism, produce circulating tumor cells, affect immune response, and, in synergy with oncogenes, produce invasive tumors that are similar to human cancers (reviewed in [27, 29, 35–42])

Are these observations relevant to human cancers? This question prompted a search for cell hybrids in human tumors.

## How to find a hybrid?

To find a hybrid in a laboratory animal, the animal is made chimeric, that is, composed of two or more genetically distinct cell populations. For example, to make two populations distinct and their hybrids easier to detect, one population is commonly modified to carry a gene encoding a green fluorescent protein, while the other a gene encoding a red protein. Hybrids are then identified as cells that carry both genes.

Chimeric animals are required for two reasons. First, unlike other features that may be specific to parental cells, nuclear genomes cannot be changed by cell fusion beyond recognition, are replicated during each cell cycle, and, consequently, can be identified unambiguously even after numerous cell divisions, which is why genomic analysis is also used in forensics to identify humans and their genealogical relationships. Second, most cells of a laboratory mouse or a human have the same genetic background because they are the progeny of one cell, the fertilized egg. As a result, cell hybrids remain “invisible” to genomic approaches unless the analyzed organism is chimeric.

**Figure 2:**
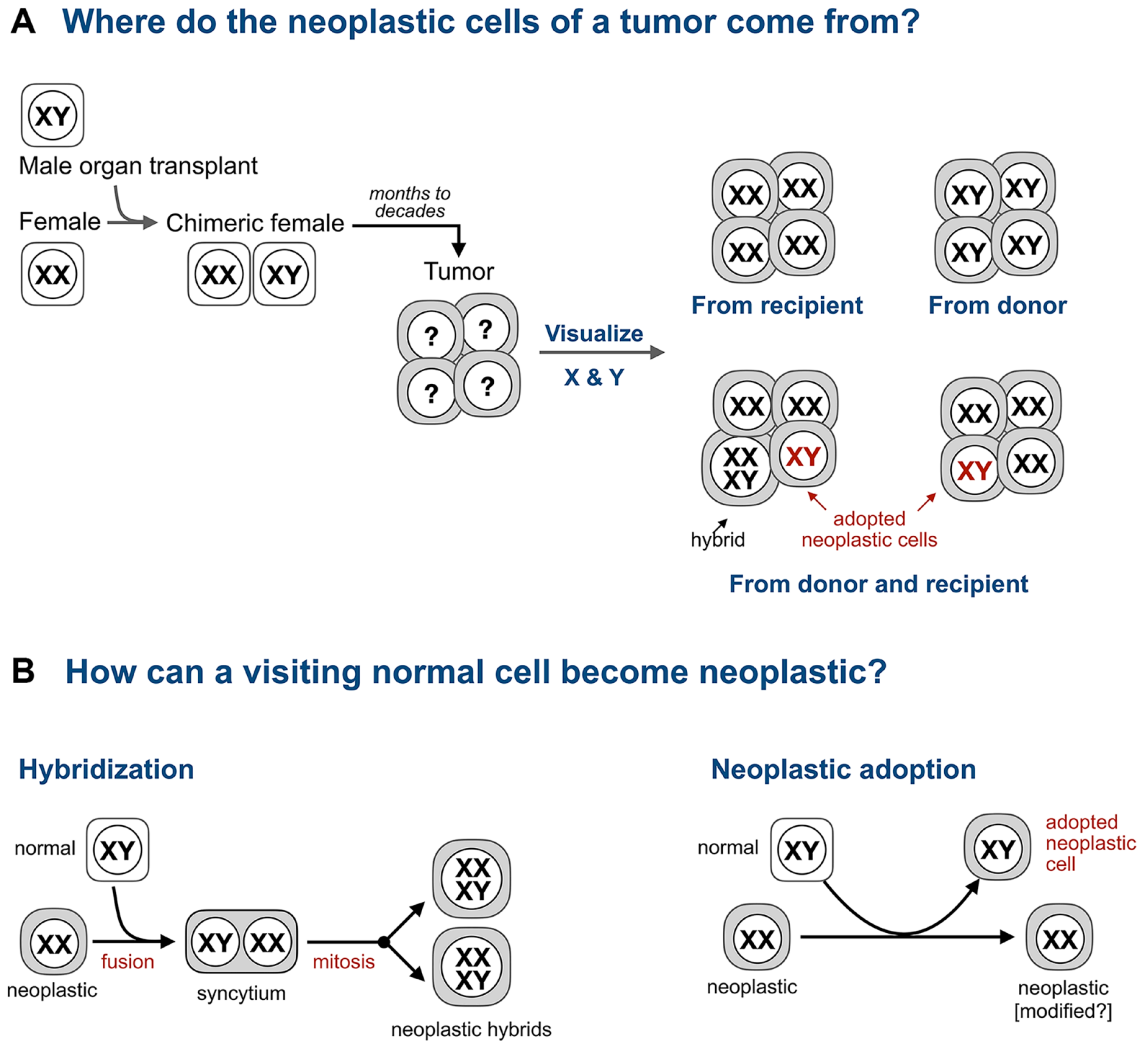
Adopted neoplastic cells in tumors from sex-mismatched transplant patients. (**A**) A typical experiment to test if tumor cells form hybrids with bone marrow derived cells in tumors from transplant patients. A female who received a bone marrow transplant from a male subsequently develops a tumor. Whether the cells of this tumor come from the donor or the recipient is revealed by visualizing sex chromosomes (XY in males, XX in females, and XXXY in predicted hybrids) and whether these cells are neoplastic or normal is determined by histopathology. (**B**) Expected intermediates for hybridization and neoplastic adoption.

Hence, the search for hybrids in human cancers turned to patients who are chimeric because prior to a cancer diagnosis these individuals received an organ transplant and, as a result, are made of two genetically distinct cell populations: one has the genome of the recipient, the other the genome of the donor. Analyzing patients who received an organ from the donor of the opposite sex, say a female who received a bone marrow transplant from a male (Figure 2), is particularly informative because the origin of individual cells (donor or recipient) in a tissue can be revealed by visualizing sex chromosomes and whether these cells are normal or neoplastic determined by histopathology.

These so called sex-mismatch patients are rare – for example, only four were found among 12,000 transplant patients by one study [43] – and so have been studies that have searched for cell hybrids in human tumors. This rarity, and the potential to reveal what had been invisible, both literally and figuratively, make the obtained results precious.

## Adopted neoplastic cells

The analysis of tissues from sex-mismatch patients has revealed two groups of tumors (Figure 2).

In the first group, which included basal cell carcinomas, skin squamous cell carcinomas, and oral squamous cell carcinomas, all of the analyzed neoplastic cells in each tumor contained the sex chromosomes of either the donor or the recipient [44–46].

This result is consistent with the current view that all neoplastic cells in a tumor are a clone of a single cell, the cell of origin [21]. Indeed, this model predicts that in a transplant patient the cell of origin can come either from the recipient, in which case all neoplastic cells of the resulting tumor should have the genome of the recipient, or from the donor, in which case all neoplastic cells should have the genome of the donor.

However, in the second group of tumors, which included lung adenocarcinoma, laryngeal squamous cell carcinoma, glioblastoma, Kaposi sarcoma, colon adenomas, esophageal carcinoma, basal cell carcinoma, squamous cell carcinoma, and pancreatic ductal carcinoma, 1% to 40%, or “a proportion” [47], of neoplastic cells identified by pathologists as “histologically malignant,” “as carcinoma cells,” “invasive,” “consistent with neoplastic colonic adenoma cells” contained the sex chromosomes of the donor, with the rest containing only the sex chromosomes of the recipient [43–45, 48–51]. Hence, each of these tumors was made of two genetically distinct populations of neoplastic cells – one derived from the recipient, another from the donor – and thus could not be a clone of one cell.

Several additional observations support this conclusion. First, not all tumors analyzed by the same approaches in the same laboratory had detectable neoplastic donor cells [45–47, 50], meaning that these cells are a feature of some tumors rather than an artifact of the methods used. Second, neoplastic donor cells were also detected among tumor cells found in the blood (circulating tumor cells, or CTC) [51], which rules out potential artifacts associated with analyzing solid tissues. Third, some of the studies verified the donor origin of neoplastic cells using multiple approaches, such as tracing a rare mutation, analyzing mitochondrial DNA, genotyping microdissected tumor cells, staining for HLA antigens, and evaluating the ploidy of donor-derived neoplastic cells by visualizing non-sex chromosomes [43, 47, 50].

The presence of donor-derived neoplastic cells could be explained, as the model which prompted the search for the hybrids had predicted, if some normal donor cells fused to the neoplastic cells of the recipient, yielding hybrids in which the neoplastic phenotype becomes dominant. However, genomic evidence for hybridization has been found only for a fraction of neoplastic donor cells and only in some tumors [48, 49, 52]. No such evidence was found [43, 45, 50] or available [44, 51] for other tumors, implying that at least some of the donor-derived neoplastic cells are not hybrids.

If they are not hybrids, then what are they?

The lack of evidence for their hybrid origin, the observation that these cells are a minority among neoplastic cells, and the finding that these cells are detected in tumors diagnosed as early as two months after an organ transplant [45] can be explained if some normal donor cells migrate into a tumor and acquire the phenotype of tumor cells without acquiring their genome, as has indeed been suggested [43, 45, 50, 53]. I will refer to such cells as adopted neoplastic cells [53], irrespective of how they become adopted, because these cells are not the genetic offspring of resident neoplastic cells.

How are these cells adopted? Who are the “adoptive parents”? What types of cells can be adopted? What is the fate of adopted cells? What kind of tumors can adopt cells? Are these cells restricted to transplant patients? To see if these questions are worth discussing, let us first consider how the presence of adopted cells would affect tumor development.

## What would the existence of adopted neoplastic cells imply?

Adopted neoplastic cells not only look as resident neoplastic cells to expert pathologists, which is by itself remarkable because how tumor cells look is used to diagnose tumors and to predict their clinical course [1], but at least some of them may have properties required to expand or seed tumors. Indeed, some donor-derived neoplastic cells were found in clusters or nests [43, 44, 48, 49], as would be expected if these cells proliferate. Whether solitary adopted cells also proliferate is unclear because migratory cells can wander away from each other instead of forming a cluster. That at least some of the adopted cells are migratory, perhaps because they retain some features of their normal precursors, follows from their presence among circulating tumor cells [30, 51, 52], an observation that also implies that adopted cells can retain their neoplastic phenotype for some time after leaving the tumor.

The ability of adopted neoplastic cells to proliferate, migrate, and retain their phenotype prompts a hypothesis that even if the majority of the adopted cells vanish, as adopting a neoplastic phenotype might trigger cell suicide, cell cycle arrest, or an attack by the immune system, some hopeful monsters, to use an evolutionary term [54] particularly fitting cancer cells, could survive, multiply, and evolve.

If so, the existence of adopted neoplastic cells would have consequences that might explain some outstanding observations.

## Tumor evolution can be genetically discontinuous

**Figure 3:**
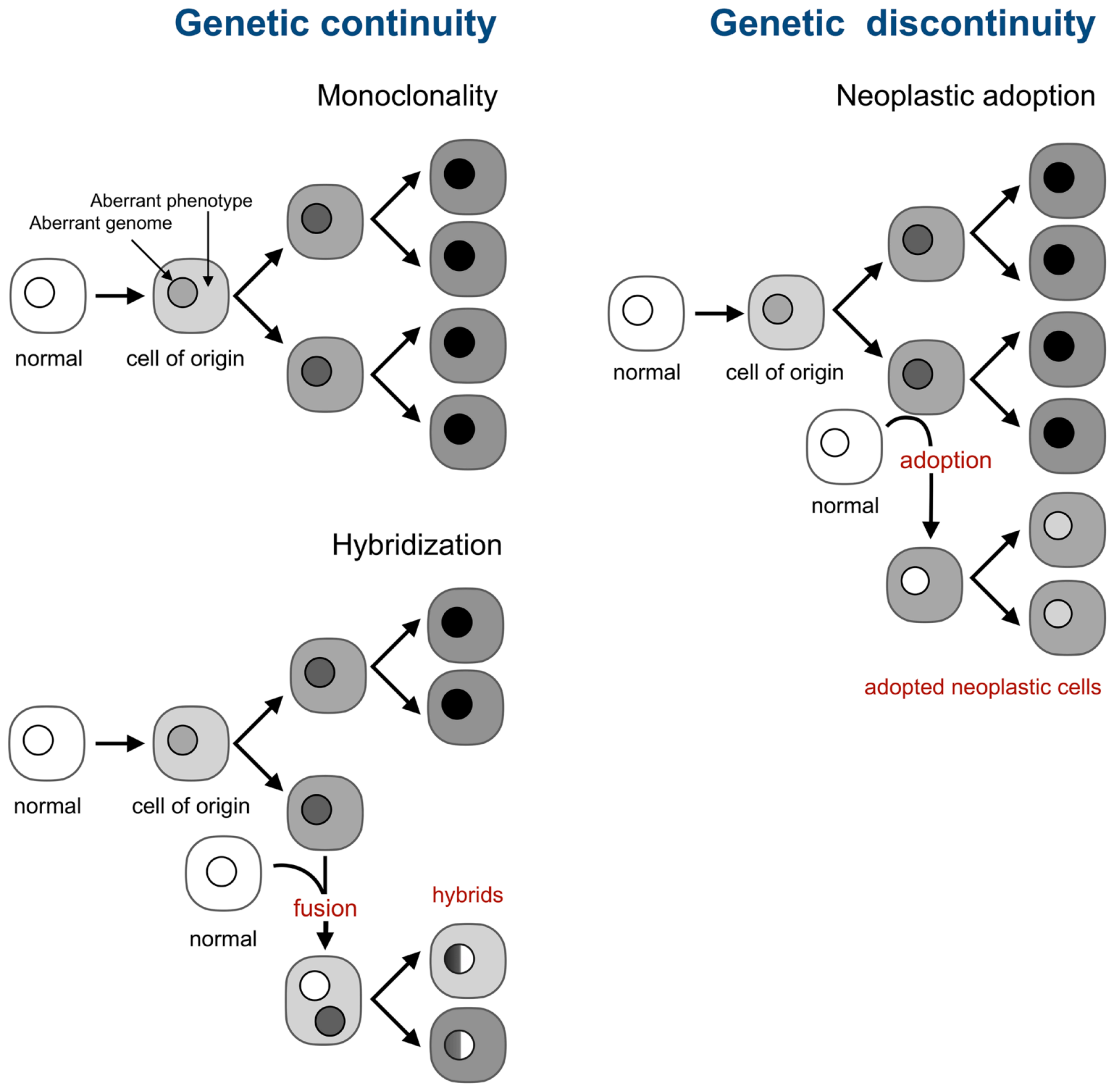
Neoplastic adoption enables genetic discontinuity in tumor development. A current view (top left) is that all neoplastic cells in a tumor are the progeny of the cell of origin, which implies genetic continuity of tumor development. This continuity holds even if tumor cells form hybrids with normal cells (bottom left). This continuity breaks, however, when a normal cell acquires the neoplastic phenotype of resident neoplastic cells without acquiring their genome (right).

A model taken by cancer research and oncology as a fact, despite contradicting evidence [55, 56], posits that all neoplastic cells of a tumor are the genetic progeny of a single cell, the cell of origin, which becomes neoplastic by acquiring genomic and epigenetic aberrations [18, 20, 21] (Figure 3). This model implies that new neoplastic cells in a tumor are produced only by the division of existing neoplastic cells, or, in other words, that tumor evolution is genetically continuous.

For example, the notion of genetic continuity underlies the effort to identify and target genomic aberrations that cause the cell of origin to become cancerous and thus are expected to be inherited by all neoplastic cells in a tumor. Hence, the hope is that targeting these aberrations would kill all tumor cells with one arrow. However, if normal cells can acquire a neoplastic phenotype without acquiring the tumor genome and thus its aberrations, targeting these aberrations is bound to fail, as has indeed been the case for reasons that are only partially understood [57–59].

This unfortunate implication of genetic discontinuity may not require many adopted cells, as a single cancer cell can seed a tumor [60], cancers can relapse in patients with undetectable residual disease [61], and metastases can emerge, apparently from single disseminated cells, years or decades after the primary tumor has been excised [62–64]. To put these facts in perspective, a tumor in which 1% of neoplastic cells are adopted could have 10^7^ of hopeful monsters per each 1 cm^3^ of the tumor [61].

Genetically discontinuous evolution can also help to explain some observations related to metastasis.

## “Lost” aberrations

All metastases are thought to be genetically continuous with a primary tumor [4]. Yet, some genomic aberrations found in a primary tumor, including deletions, can be absent in its metastases [4, 62, 65–67].

How are these aberrations lost?

**Figure 4:**
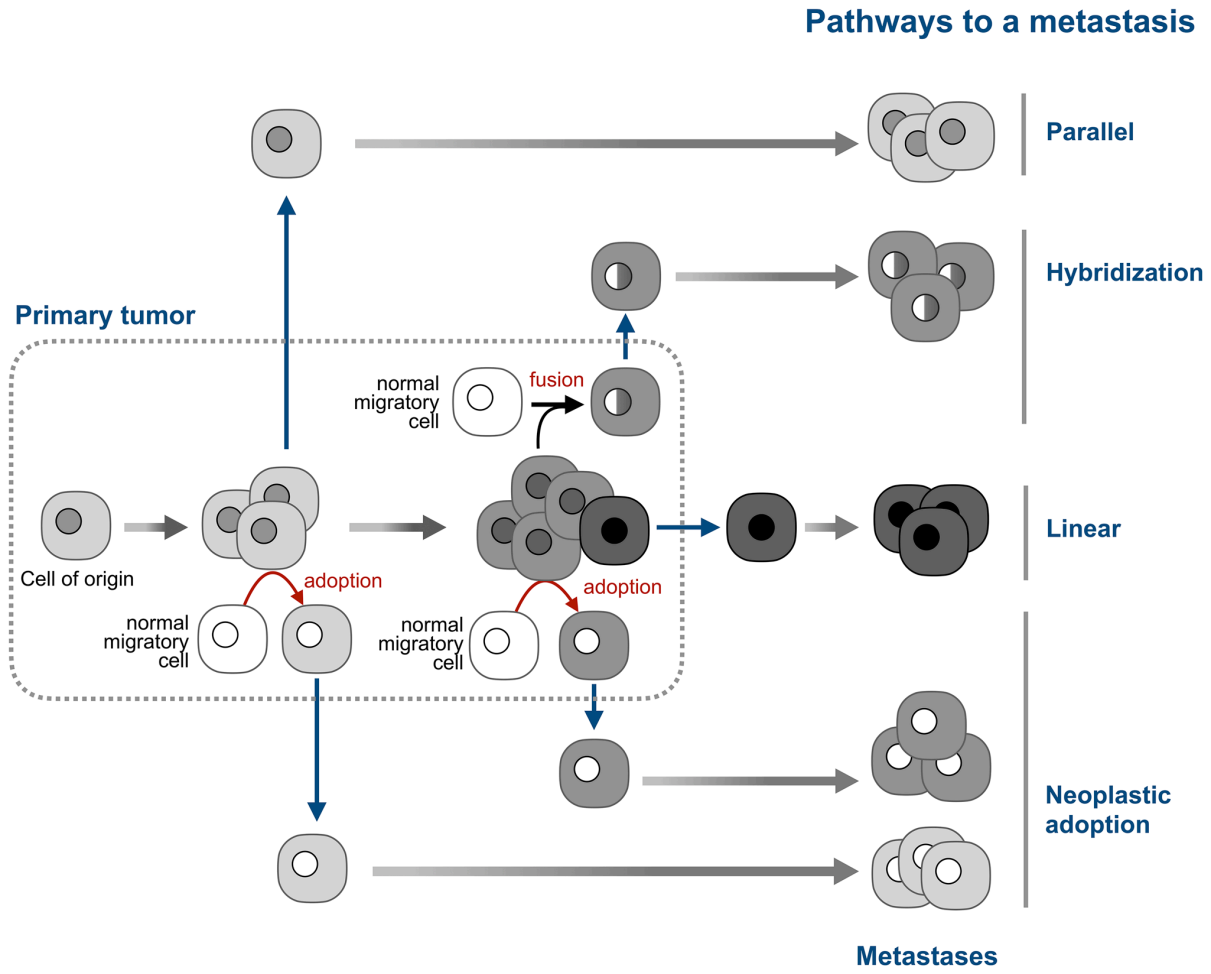
Neoplastic adoption as a pathway of metastasis. The prevailing linear model, posits that tumor cells become able to seed new tumors by accumulating additional mutations late in tumor development. The parallel model posits that tumor cells disseminate early in tumor development and then continue to evolve in parallel with the primary tumor. The hybridization model argues that metastases are formed by hybrids between tumor cells and normal migratory cells. The neoplastic adoption model suggests that metastases can be seeded by normal migratory cells that acquire a neoplastic phenotype of tumor cells without acquiring their genome. Both hybridization and neoplastic adoption models suggest that metastatic cells can be formed at any stage of tumor development. Note that these four models are not mutually exclusive.

The parallel model of metastasis [62] explains this paradox by positing that neoplastic cells that seed metastases leave the primary tumor early in its development, before the “lost” aberrations appear (Figure 4). The tumor and the “seeds” then evolve in parallel, with the potential of convergent genomic evolution characteristic to the cells of the same cell type [68]. This model can explain why single breast cancer cells found in bone marrow have few or no chromosomal aberrations which are abundant in primary tumors [62, 69], and why metastases can be present with no detectable primary tumor [70].

Adopted neoplastic cells would also seed metastases that “lost” the aberrations of the primary tumor, but for a different reason – because adopted cells do not inherit the tumor genome. This difference also means that adopted cells can seed metastases with “lost” aberrations at any time of tumor development, not only at its early stages, as the parallel model suggests.

## A leap

The failure to find mutations that cause metastasis prompted a search for a “discrete step in tumor evolution that may be independent of specific oncogene pathways or mutations and instead co-opts cellular traits that mitigate immunologic, genotoxic and therapeutic stressors accreted during tumorigenesis.“ [5]. In evolutionary biology such steps are known as saltational (from the Latin for *leap*), as they suddenly (on the evolutionary time scale) enable “profound phenotypic novelties or species” [54].

For example, the metastasis seed preselection model proposes that neoplastic cells become “bone marrow-like” under the influence of bone marrow-derived cells, which are abundant in tumors, and thus can prosper and proliferate once they reach the bone marrow, a common site for metastasis [71, 72]. The existence of adopted cells derived from bone marrow transplants [43, 45, 51] implies that the influence can flow in the opposite direction as well, to make some bone marrow-derived cells neoplastic.

Several properties of neoplastic adoption make it a suitable candidate for the sought after “leap”. First, this phenotypic switch is a discrete step and it happens suddenly on the time scale of tumor development. Second, adopted cells do not inherit the burden of genomic aberrations, and the consequent aberrant antigens targeted by the immune system [73], because they do not inherit the tumor genome. Finally, retaining some properties of normal migratory cells [51] enables safe passage to distant organs and helps to settle there.

## Puzzling circulating tumor cells

Circulating tumor cells (CTC), discovered a century and a half ago [74], are neoplastic cells present in the blood of patients with solid tumors and are thought to include the precursors of metastases [75].

Paradoxically, a fraction or, in some cases, most of CTC released by some non-hematological tumors, such as melanoma, breast, ovarian, and pancreatic cancers, were found to carry CD45, a protein whose expression is normally restricted to bone-marrow derived cells [30, 51, 76–78]. Hence, these cells (CTC-CD45) were viewed as a persistent artifact until an appeal to give them a closer look [77] revealed that they indeed exist [79] and that their concentration inversely correlates with the survival of patients with pancreatic cancer [30, 51].

The presence of a lymphocyte protein on the cells of non-hematological tumors prompted a hypothesis that CTC-CD45 are hybrids between tumor cells and leukocytes [79]. As a result, these cells have been reported as macrophage-tumor cell fusions [80], circulating hybrid cells [30], or simply hybrids [78]. However, genomic evidence for the hybrid origin of these cells is still unavailable, leaving open other explanations: that CD45 is present due to aberrant gene expression, which is common in cancer, or that cancer cells acquire this protein through trogocytosis [81], a process that enables the intercellular exchange of membrane proteins [82], or by a similar phenomenon termed vampirization [83].

The existence of adopted cells suggests another potential explanations: that some CTC-CD45 are bone marrow derived cells that adopted a neoplastic phenotype. This explanation is consistent with a report that only a fraction of CTC-CD45 recovered from a pancreatic cancer patient have a mutation characteristic to this cancer [52]. Given that only one of 10,000 CTC is estimated to seed a metastasis [74], adopted cells may be a candidate for this subpopulation.

## What kind of cells can be adopted?

The fact that various normal cells are commonly present in tumors means that only some of these cells can be adopted. What type of cells could they be?

Adoptable cells should be migratory to enter a tumor, able to adopt the phenotype of surrounding cells, and capable of residing in more than one organ, as adopted cells were detected in patients transplanted with all transplanted organs that were analyzed: bone marrow [43, 45, 49, 51], mobilized peripheral blood stem cells [45], and kidney [44, 47, 50].

This profile matches that of mesenchymal stem cells (MSCs), a group of migratory cell types that are found in bone marrow and some other organs, are capable of assuming a variety of phenotypes, and are prone to home to damaged tissues and tumors [84–87]. Since MSCs transplantation, including genetically modified and allogeneic cells, has been used in more than 1,000 clinical trials [87], the treated patients can be used to test whether MSCs contribute to the neoplastic population of tumor cells.

While MSCs are a suitable candidate for a cell type susceptible to adoption, the spectrum of cell types that can be adopted will need to be determined. These studies can be informed by considering potential mechanisms of adoption.

## How can a normal cell adopt a neoplastic phenotype?

**Figure 5:**
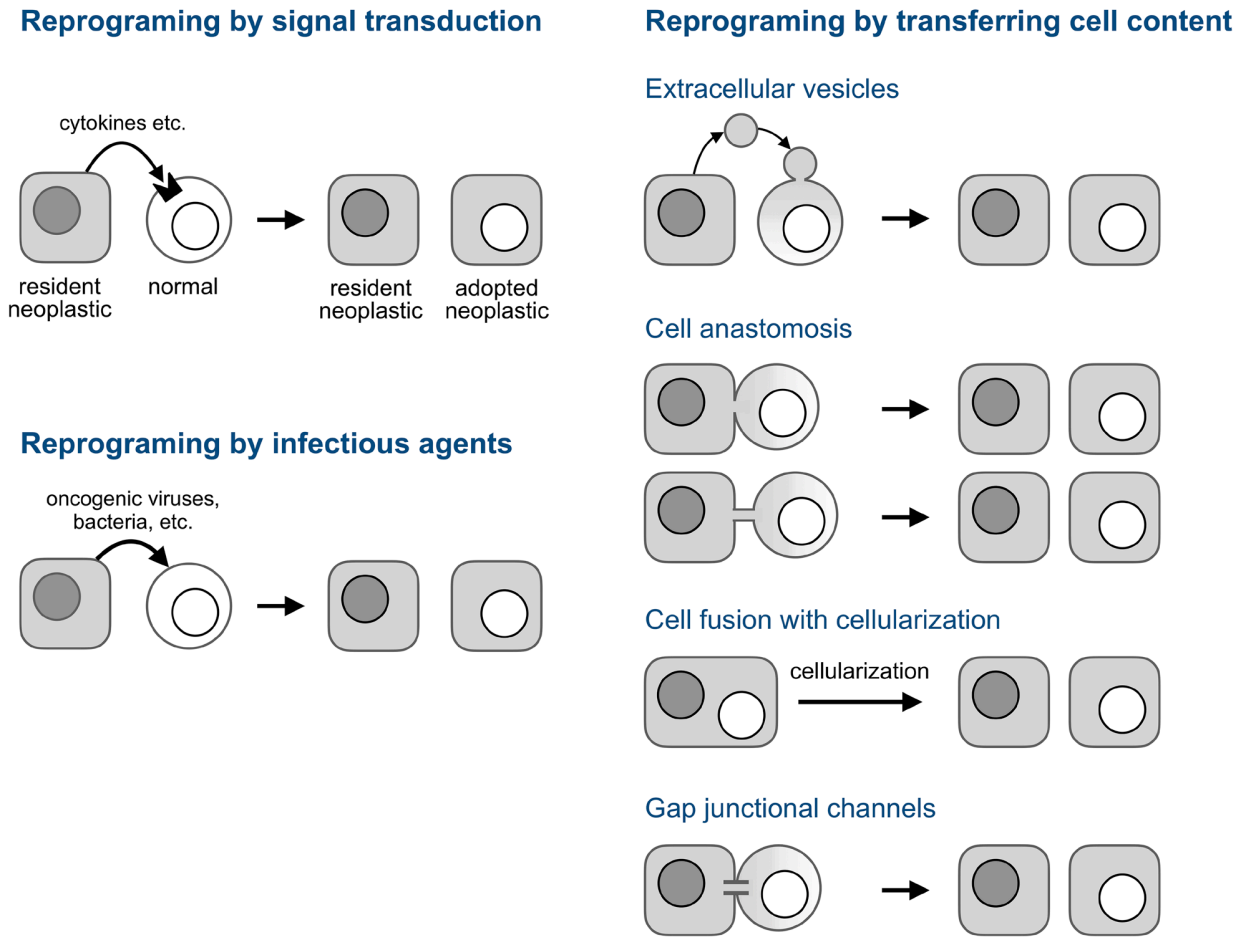
Potential mechanisms of neoplastic adoption.

The lack of genetic evidence for hybridization prompted a hypothesis that cells derived from bone marrow can adopt the phenotype of resident tumor cells by “subjection to locally released growth factors and cell-cell contact,” a mechanism named developmental mimicry [45] (Figure 5, left panel). However, this hypothesis poses a conundrum: How can a normal cell mimic a neoplastic phenotype by responding to external signals if, as the prevailing view of cancer posits, this phenotype results from genomic aberrations which a normal cell lacks by definition? This puzzle suggests two, not mutually exclusive, solutions.

One, that at least some neoplastic phenotypes are caused not by genomic aberrations. For example, they may be caused by aberrations in molecular and electric processes that organize cells into a tissue and determine their phenotypes [88–90]. If so, then some normal cells entering a tumor could be made neoplastic by the same forces and mechanisms that created resident neoplastic cells in the first place.

Another explanation is that a neoplastic phenotype, whatever its origin, can be transmitted from a tumor cell to a normal cell, or, in other words, that a cancer cell can make a normal cell cancerous. This century-old concept [91] was rediscovered four decades ago as horizontal oncogenesis to explain how a human adenocarcinoma transplanted into a mouse could induce a *mouse* sarcoma, a tumor which arises from a different cell type, in the adjacent connective tissue [92, 93]. Two mechanisms of transmission were initially considered: an oncogenic infectious agent and fusion between the transplanted and host cells that results in neoplastic hybrids. As we have discussed, such hybrids have indeed been documented, but so have been grafted human cancers that induced mouse tumors of the same or different type without any evidence for hybridization [94, 95].

In the absence of an infectious agent, these observations and neoplastic adoption can be explained if transcription factors and other molecules that determine a neoplastic phenotype are transmitted between cells without forming cell hybrids. Several mechanisms can do that (Figure 5, right panel).

Extracellular vesicles are membrane-enclosed cell fragments of various origin, content, and size that are released by cells and can deliver their content to a target cell by fusing to its plasma membrane [96–98]. This transfer has been implicated in various aspects of cancer development [98–100] and in the transfer of neoplastic properties to normal cells in particular.

For example, vesicles isolated from human colorectal cancers induced “tumor-like morphological changes and marked growth rate increase” in human mesenchymal stromal cells isolated from normal colon [101]. Likewise, vesicles derived from human prostate cancer cell lines enabled normal adipose stem cells isolated from prostate cancer patients to form neoplastic lesions “that were grossly and histologically comparable with those developed by [prostate cancer] cells” [102], while vesicles from a breast cancer cell line enabled an immortalized breast epithelial cell line to form tumors [103]. These findings are consistent with earlier results obtained by using cytoplasts, cell fragments made by enucleating cells and similar in size to some naturally occurring extracellular vesicles [96]. Fusing cytoplasts from tumorigenic cells with normal human lymphocytes produced immortalized cell lines which “exhibited morphological diversity ranging from adherent cells to free floating round cells” [104], while fusing cytoplasts from a tumorigenic breast epithelial line to its non-tumorigenic predecessor yielded tumorigenic cells [105]. In essence, extracellular vesicles and cytoplasts produced adopted neoplastic cells in the dish.

However, the oncogenic effects of extracellular vesicles have been questioned, in part because the resulting phenotypes were transient in an experimental system [106] and their persistence “would violate several tenets of the existing cancer progression paradigm” [107], as if some of these tenets were not contradicted by facts, logic, and clinical outcomes [108–113]. Hence, “the possibility that horizontal transfer of oncogenic material [by vesicles] can lead to tumor formation is the subject of considerable debate” [99]. The evidence for the presence of adopted neoplastic cells in human cancers suggests that this debate is no longer only academic, especially because extracellular vesicles are not the only mechanism for transferring cellular components nor is it most efficient.

While extracellular vesicles transfer cell content in small packets, cell anastomosis (Figure 5) bridges cells directly through pores (anastomoses) made by merging the plasma membranes of adjacent cells [114–118]. These pores, which range from 100 nm to a few microns [115], can bridge adjacent cell bodies, in which case the connection has been called partial [115] or transient [119] cell fusion, or form anastomosis tubes by anastomosing a protrusion of one cell with the body of another, or two protrusions to each other.

Protrusions that transmit “dyestuff, mitochondria and granules from one cell to another” [120] had been reported a century ago [120, 121] and were rediscovered more recently [122, 123] as a family of intercellular tubes with diverse properties, functions, and names that enable cells change the properties of other cells by transferring cellular components [122–127].

Unlike cell fusion, which combines the entirety of two cells into one morphologically and functionally distinct unit, a syncytium, which can then produce hybrids, cell anastomosis enables the transient, localized, and regulated sharing and exchange of cellular components, including whole nuclei [128–130], while preserving the morphology of the bridged cells. As a result, anastomosis can be overlooked even in experimental systems by approaches that focus on detecting cell hybrids, as no hybrids or syncytia are formed. Anastomoses can be visualized by electron microscopy [114–118] and their presence detected by methods that register the consequences of transient cell bridging, such as DNA modifications made by the Cre/loxP system [119, 131] or mitochondria transfer. For example the finding that a transmissible canine cancer has acquired mitochondrial but not nuclear genomes from several of its hosts [132] can be explained by anastomosis.

Cell anastomosis is a candidate for processes that enable neoplastic adoption because mesenchymal stromal cells are prone to anastomose with other cells [133, 134] and because anastomoses, along with other intercellular bridges, tie neoplastic and normal cells of human cancers into a network, a process whose significance has been increasingly appreciated [125, 129, 130, 135–139]. Adopted neoplastic cells might emerge as a part of this process.

Cell fusion may also be involved in neoplastic adoption if syncytia it forms undergo cellularization, an enigmatic but well documented process by which multinucleated cells split into mononuclear cells without entering mitosis. Cellularization is common in protists [140], is a required part of *Drosophila* development [141], is involved in tissue regeneration in the newt [142], and also happens in mouse osteoclasts [143], myotubes [144, 145], and, under the name of neosis, in transformed multinucleated mouse cells [146]. Some small molecules that induce cellularization in the newt also do so in mouse cells [142] implying that the mechanisms of cellularization are conserved among species.

While extracellular vesicles, anastomosis, and cell fusion use membrane fusion to transfer cellular components, *gap junctional channels* enable this transfer by piercing the membranes of adjacent cells. These transmembrane protein complexes can transmit metabolites smaller than 1.5 kDa, of which a cell has about forty thousand [147], regulatory RNAs, and some proteins [148–151]. The role of this transfer in cancer has been studied for half a century to implicate it in nearly all aspects of this disease [147]. However, these channels might contribute to neoplastic adoption not only by transferring molecules but also by functioning as “biological transistors” [152, 153] which enable bioelectrical signaling between non-neuronal cells, a process implicated in cancer [90, 154–156].

Overall, several mechanisms, including infectious agents, “classic” signaling between autonomous cells, tissue organization fields, and intercellular component transfer can potentially explain how a normal cell can acquire the neoplastic phenotype of surrounding cells. I find component transfer intellectually attractive because transferring activities and structures that determine a phenotype can readily explain how this phenotype, normal or abnormal, can be imposed on another cell. This mechanism can also explain how the imposed and suppressed phenotypes can blend at various ratios, and how this blending can produce emergent properties [32, 157].

## What components need to be transferred to induce a neoplastic phenotype?

This question is difficult to answer definitively because how a cell becomes neoplastic and how its phenotype is maintained is still a matter of debate. However, the bridging mechanisms that we have discussed (Figure 5) can transfer practically any component mentioned in this debate, from oncogenes to oncometabolites [158, 159].

For example, the activity responsible for the ability of cytoplasts from tumorigenic cells to immortalize human lymphocytes [104] was identified as two short species of endogenous cytoplasmic DNA [160]. Cytoplasmic DNA includes retrotransposons, linear and circular chromatin fragments of various size, mitochondrial DNA, and micronuclei [161], all of which are transferrable by anastomosis [162] or by extracellular vesicles [163–165].

Micronuclei are of particular interest because they encapsulate chromosomes and their fragments and can rearrange them by a process called chromothripsis, which breaks chromatin into fragments and then stitches them in apparently random order [166]. Besides other consequences, this process can yield circular extrachromosomal DNA (ecDNA) [167], which was found in nearly half of human cancers, “almost never found in normal cells,” and affects tumor evolution and drug resistance presumably by harboring amplified oncogenes [168–170].

Transferring tumor mitochondria to a normal cell by anastomoses or extracellular vesicles would transmit mutations in mitochondria DNA that have been considered oncogenic [171], and also has the potential to make the target cell neoplastic by reprograming its metabolism [172], with the concomitant production of oncometabolites, which are metabolites that deregulate gene expression if present at an increased concentration [158, 159]. Given their size, oncometabolites can also be transferred by gap junctional channels.

Likewise, merging plasma membranes by anastomosis or transferring membrane fragments by extracellular vesicles enables the migration of membrane-associated molecules, including growth factor and cytokine receptors implicated in causing and maintaining cancer phenotypes [58].

Overall, multiple components that are known to be transferred between cells can contribute to neoplastic transformation. Learning which of these components enable neoplastic adoption and whether this process involves component transfer at all may be helped by knowing the reasons for neoplastic adoption.

## Why are normal cells converted into neoplastic?

**Figure 6:**
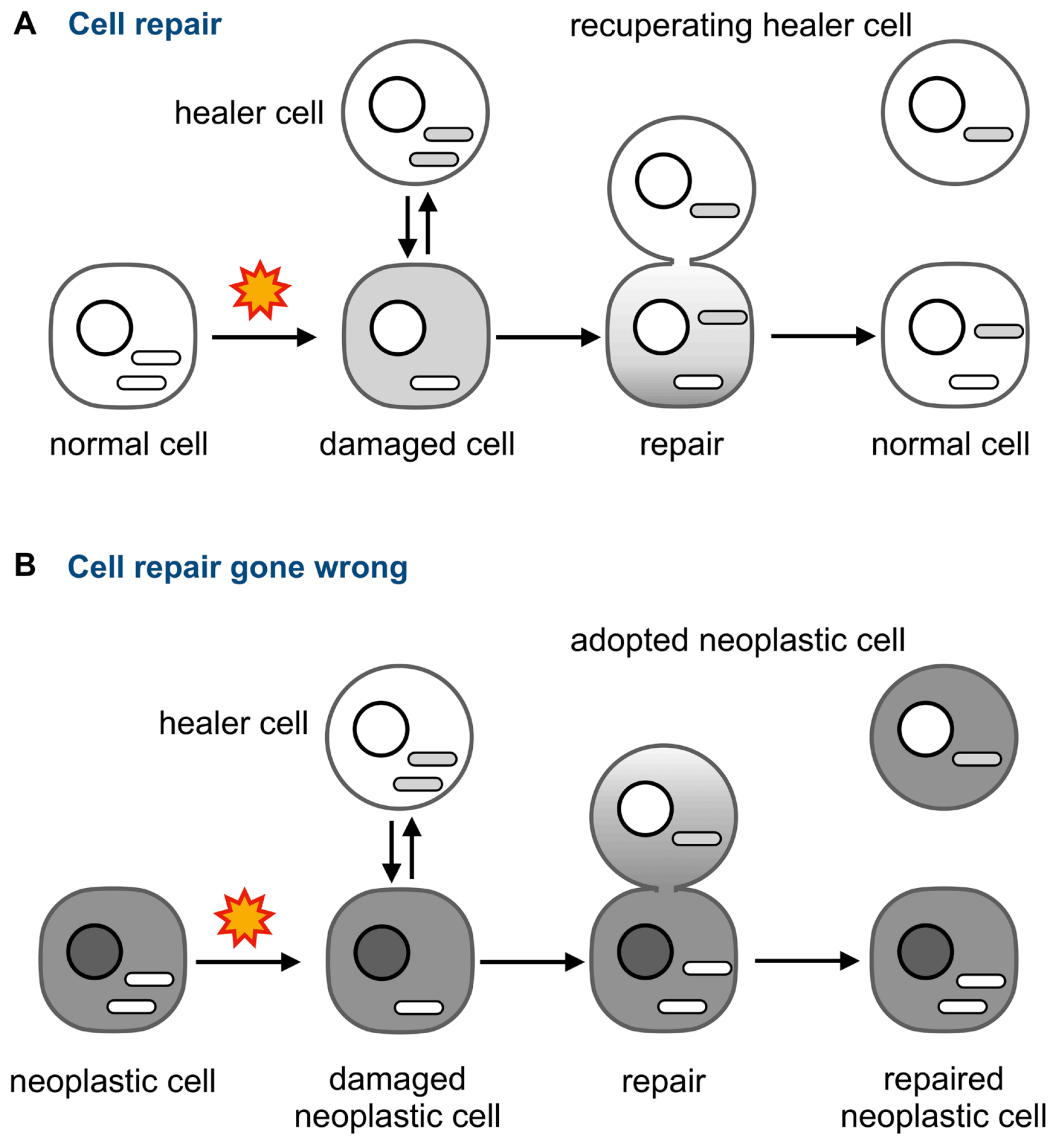
Neoplastic adoption as a side effect of cell repair. (**A**) An injured cell whose mitochondria and other cytoplasmic components are damaged can receive them from an intact “healer” cell. The transfer is unidirectional to make sure the damaged components are not transferred from the “patient”. (**B**) If the injured cell is neoplastic, the transfer may become bidirectional because multiple functions of the neoplastic cells are deregulated. As a result, regulatory molecules that maintain a neoplastic phenotype are transferred to the healer and change its phenotype into neoplastic. Note that the repaired neoplastic cell may also change its properties by acquiring the content of the healer.

One possibility is that neoplastic adoption illustrate the notion that no good deed goes unpunished (Figure 6).

Tissue injury, such as irradiation or chronic inflammation, prompts circulating bone marrow-derived cells to fuse to damaged cells [173, 174] or to repair them by delivering molecules and organelles through anastomoses [123, 124, 134, 175–177]. These observations pointed to the existence of a cell repair mechanism that heals injured cells by providing intact components, and mitochondria in particular [123]. This mechanism has been already explored as a therapeutic approach using MSCs as “healer” cells, even though the underlying molecular mechanisms and possible side effects are yet to be understood [87, 134, 178].

For example, what would happen if the damaged cell is neoplastic? Can the “healer” cell contract the neoplastic “disease” through the same anastomosis tubes that deliver the “cure,” perhaps because some abnormalities of the neoplastic cell affect how the two cells are bridged, what components are transferred, and in which direction? For example, cytoskeleton, which is deregulated in cancer [179], is involved in forming intercellular bridges [180], determines what these bridges transmit [181], and regulates the size of pores formed by the fusion of plasma membranes [182].

The possibility of contracting a neoplastic phenotype as a side effect of repairing a neoplastic cell can explain why adopted cells were detected not in all tumors, as a cell would be adopted nor merely because it wanders among neoplastic cells but because it attempts to repair them. If so, the incidence of neoplastic adoption in a tumor would depend on the extent of genomic and other aberrations, hypoxia, inflammation, infection, or other internal and (micro) environmental factors that can result in cell damage.

The model that neoplastic adoption is a side effect of cell repair, and the observation that adopted cells are present in colon adenomas [45], which are benign tumors that can progress to cancers, suggest that non-cancerous neoplastic lesions can progress to cancerous by adopting migratory cells. This hypothesis can explain why cancers can arise without a detectable precursor lesion, as happens with the majority of melanomas [183] and some types of lung cancer [184]. For example, this phenomenon would be expected if some cells are adopted by a microscopic neoplastic lesion, which are by far more abundant than cancers [185, 186], and then evolve on their own, locally or elsewhere, even if the adopting lesion vanishes.

This model would also be consistent with the exponential increase of cancer incidence with aging, a process associated with accumulating cellular damage of various kind [187], and the fact that chronic inflammation, which is associated with the recruitment of bone marrow derived cells to abnormal tissues, increases the incidence of cancer [188, 189].

Finally, the model that neoplastic adoption is a consequence of repair implies that a treatment that damages cancer cells without killing them can create adopted neoplastic cells. These cells may take revenge even if the “adopting parents” eventually die, reminding us, to rephrase a quote, that if you strike at an emperor, you must kill him [190, 191].

Another possibility is that neoplastic adoption is not a side effect of cell repair but results from the propensity of neoplastic cells to bridge neoplastic and normal cells into the networks using anastomosis tubes and gap junctional channels, as has been documented in glioblastoma [129, 130, 139, 192, 193].

Both the side effect model and the network model imply that neoplastic adoption may affects the “adoptive parents,” for example if the unidirectional transfer of components from the “healer” to the “patient” turns into intercellular exchange. If so, then neoplastic adoption not only would produce adopted neoplastic cells but would also modify resident neoplastic cells. For example, resident cells can become migratory or “invisible” to the immune system. If so, neoplastic adoption would further increase the diversity of cell types in a tumor and thus its ability to preempt our assassination attempts when they are still on the drawing boards of pharmaceutical companies.

## Are adopted neoplastic cells present in non-transplant patients and how can these cells be detected?

Adopted cells have been revealed by analyzing tumors from transplant patients. Because these individuals have a medical history that differs from that of most cancer patients, it is reasonable to ask if adopted cells are also present in the tumors of non-transplant patients and how these cells can be detected.

A hint that neoplastic adoption is not limited to transplant patients comes from the studies of fetomaternal microchimerism, a condition in which cells exchanged between a woman and her child during pregnancy persist in their bodies, sometimes for decades [194–196]. If the fetus is a male, fetal cells can be identified in the mother’s tissues by visualizing the Y chromosome, the same approach that was used to look for cell hybrids in transplant patients.

This approach has revealed that some fetal cells have properties of migratory progenitors with multilineage differentiation capacity [197, 198]. Like mesenchymal stromal cells, these progenitors home to damaged organs and tumors, become morphologically “undistinguishable from the surrounding cells in terms of size and nuclear shape“ [199], and begin to express antigens specific to the host tissues, whether normal or neoplastic [196, 199–202].

Yet, fetal cells that have the features of surrounding tumor cells have not been considered neoplastic because these cells were not found in clusters “even if they expressed antigens that can be found within the adenocarcinoma“ [199]. However, some fetal cells in cervical cancers were found in clusters [200], and one can argue that some proliferating cells would not be expected to form clusters if these cells are migratory. Hence, a suggestion that fetal cells can “adapt a malignant phenotype and potentially fuel tumorigenesis” [196].

The similarities between fetal progenitors and mesenchymal stromal cells in their ability to home to damaged tissues and adopt the phenotype of the resident cells suggests that fetal cells could be “tracers” of adult cell populations that have similar properties, including the ability to adopt a neoplastic phenotype.

The uncertainty about whether fetal cells can be neoplastic, as well as about some of their other properties, stems from their rarity – a few to a hundred are found per million of maternal cells analyzed [199] – although this incidence may be underestimated [196]. However, given the increasing sophistication of automatic tissue analysis [203] this rarity may be compensated by the abundance of microchimeric cancer patients, as the majority of women have fetal cells even if their pregnancy was incomplete or went unnoticed [198, 204].

Pregnancy provides another, yet to be explored, opportunity – to detect adopted neoplastic cells and cell hybrids by analyzing gestational tumors. Gestational tumors, which include moles and gestational choriocarcinoma, arise from the cells of the conceptus and thus are genetically distinct from the cells of the mother [205]. Hence, adopted cells, as well as hybrids between neoplastic and normal cells, can be detected by the approaches applied to transplant patients [53], especially because some gestational tumors have only a male genome, which facilitates the analysis. Another advantage of analyzing gestational tumors is the ability to trace normal cells derived from all organs of the patient rather than only from a transplanted organ.

To look beyond chimeric tumors, the concept of neoplastic adoption predicts that some histologically neoplastic cells of genomically aberrant tumors should have a normal genome, and that the offspring of these cells evolves in parallel with the resident cells of the tumor. This prediction can be tested by comparing the genomes and histopathology of single cells from non-chimeric tumors. Given precedents with cell types, such as CTC-CD45, that were neglected because they were not supposed to exist [79], it might well be that adopted cells have been put aside as outliers that are too unusual to consider while searching for something anticipated.

Finally, as experiments with humans can go only that far, and fortunately so, testing the hypotheses we have discussed will require experimental systems, such as human tumor explants which have been explored to reveal intercellular bridging [129, 130] and chimeric animals designed to monitor cell fate, cell fusion, and component transfer [206]. Keeping in mind that this transfer can be mediated by more than one mechanism and that intercellular signaling that does not involve component transfer may also be involved can help to use these systems to their full potential. The information obtained from existing and new experimental systems may suggest new approaches for detecting adopted cells in human tumors and how to use these cells for clinical needs.

Meanwhile, I hope that considering and testing the concept of adopted neoplastic cells will prove to be useful in explaining puzzling observations related to neoplasia and would lead to ideas, discoveries, and technologies beneficial to future cancer patients.
